# Targeting of highly conserved Dengue virus sequences with anti-Dengue virus *trans*-splicing group I introns

**DOI:** 10.1186/1471-2199-11-84

**Published:** 2010-11-15

**Authors:** James R Carter, James H Keith, Pradip V Barde, Tresa S Fraser, Malcolm J Fraser

**Affiliations:** 1 Eck Institute for Global Health, Department of Biology, University of Notre Dame, Notre Dame, IN, USA 46556; 2 Department of Biomedical Sciences, GeNYsis Center for Excellence in Cancer Genomics, University at Albany, Rensselaer, NY, USA 12144; 3 National Institute of Virology, 130/1 Sus Road, Pashan, Pune - 411021, Maharashtra, India

## Abstract

**Background:**

Dengue viruses (DENV) are one of the most important viral diseases in the world with approximately 100 million infections and 200,000 deaths each year. The current lack of an approved tetravalent vaccine and ineffective insecticide control measures warrant a search for alternatives to effectively combat DENV. The *trans*-splicing variant of the *Tetrahymena thermophila *group I intron catalytic RNA, or ribozyme, is a powerful tool for post-transcriptional RNA modification. The nature of the ribozyme and the predictability with which it can be directed makes it a powerful tool for modifying RNA in nearly any cell type without the need for genome-altering gene therapy techniques or dependence on native cofactors.

**Results:**

Several anti-DENV Group I *trans*-splicing introns (αDENV-GrpIs) were designed and tested for their ability to target DENV-2 NGC genomes *in situ*. We have successfully targeted two different uracil bases on the positive sense genomic strand within the highly conserved 5'-3' cyclization sequence (CS) region common to all serotypes of DENV with our αDENV-GrpIs. Our ribozymes have demonstrated ability to specifically *trans*-splice a new RNA sequence downstream of the targeted site *in vitro *and in transfected insect cells as analyzed by firefly luciferase and RT-PCR assays. The effectiveness of these αDENV-GrpIs to target infecting DENV genomes is also validated in transfected or transformed Aedes mosquito cell lines upon infection with unattenuated DENV-2 NGC.

**Conclusions:**

Analysis shows that our αDENV-GrpIs have the ability to effectively *trans*-splice the DENV genome *in situ*. Notably, these results show that the αDENV-GrpI 9v1, designed to be active against all forms of Dengue virus, effectively targeted the DENV-2 NGC genome in a sequence specific manner. These novel αDENV-GrpI introns provide a striking alternative to other RNA based approaches for the transgenic suppression of DENV in transformed mosquito cells and tissues.

## Background

The mosquito-borne Dengue viruses (DENV) are responsible for approximately 100 million infections and 200,000 deaths each year with 2.5 billion people remaining at risk for DENV infection, making DENV one of the most important viral diseases in the world (1). Infection with one of four antigenically distinct, but related Dengue virus serotypes (designated DENV 1 through 4) can result in Dengue fever (DF) and/or Dengue hemorrhagic fever (DHF) [1]. DF and DHF are endemic to tropical and subtropical regions of the world, but global changes in climate, rapid dispersal of virus due to ease of global travel, and migration of humans to non-tropical regions has resulted in DENV outbreaks in areas that were once non-endemic to the Dengue viruses [2,3]. Modern travel and shipping inevitably leads to an increase in the number of cases in developed countries as well, including a recent outbreak in the Hawaiian islands in 2001 (Source: CDC). These viruses are maintained in a cycle that involves humans as well as the dipteran *Aedes aegypti *mosquito which preferentially feeds on human blood and is widely distributed throughout the world [2,3].

The current lack of an approved effective tetravalent vaccine and the ineffectiveness of insecticide control measures continue to warrant a search for alternative strategies to effectively combat DENV. Newer approaches that have received considerable attention include interference with the extrinsic incubation cycle of DENV replication within the arthropod vector [2,3]. One such approach envisions population replacement of vector competent mosquitoes with those refractory for infection and/or transmission of the virus, which could theoretically halt disease transmission [2,3]. This approach has distinct advantages for environmental safety, cost effectiveness, and long term disease suppression.

Our lab has been exploring anti-DENV ribozyme strategies for intracellular suppression of virus infection as a means of transgenic immunization of mosquitoes. In a previous report we examined the effectiveness of hammerhead ribozymes in suppressing DENV infection in retrovirus transduced mosquito cells [4]. While we identified several ribozymes that are effective in significantly reducing DENV 2-NGC infection of *Ae. albopictus *C6/36 cells, our inability to target sequences that are conserved among all serotypes require investigation of additional ribozymes with potential for wider specificity. As an alternative strategy we are investigating the feasibility of utilizing a Group I intron *trans*-splicing strategy to target highly conserved sequences within the DENV genome.

The *trans*-splicing reaction of the Group I intron is derived from the natural cis-splicing reaction. Both the cis and *trans*-splicing reaction can be divided into two distinct successive transesterification steps [5]. The primary difference between the two reactions is that while the cis-splicing reaction occurs along one continuous RNA molecule to join a 5' and a 3' exon, the *trans*-splicing intron is located on the same molecule as the 3' exon, but seeks out a separate 5' exon to which it can append the 3' exon [6].

The engineered *trans*-splicing activity of the Group I intron is a versatile tool with respect to the 'editing' of RNA [7-17]. Group I intron *trans*-splicing has been used in a number of applications, such as: repair of mutant α-globin mRNA [8], restoration of wild-type p53 activity in three cancerous cell lines [18], re-establishment of the function of the canine skeletal muscle chloride channel [19], and induction of p16 activity in a pancreatic cell line [10]. More applicable to our research is the *trans*-splicing group-I intron targeting of the HIV-1 *tat *[20], cucumber mosaic virus coat protein mRNAs [7], and the hepatitis C virus internal ribosome entry site (HCV-IRES) [21].

Group I introns are subject to the same limitations as antisense or RNAi methods of RNA suppression because the high mutation rate of the DENV genome promotes the spread of strains capable of avoiding the antisense recognition essential to the *trans*-splicing reaction. Approaches that inhibit DENV infection by direct interaction with the RNA genome must be designed to act upon invariant sequences to be effective. The most invariant segments of the DENV genome are the 5' and the two 3' cyclization sequences (5'CS, CS1, and CS2 respectively) which are involved in the formation of a panhandle structure that is apparently essential for genome replication [22,23]. These cyclization sequences are separated by such a large intervening length of RNA that they are effectively acting in a *trans *manner, and since they are able to base-pair with each other their secondary structure is likely open and conducive to base-pairing.

The 5'CS is located downstream of the polyprotein start codon, well within the ORF of the Capsid (CA) protein. The stringency of tolerable mutations in this sequence may be increased by the need of the virus to conserve a functional CA protein. In fact, all mosquito-borne flaviviruses share an 8 bp stretch of nucleotides within this 5' CS sequence [24].

In this study we designed Group I introns to target and catalyze *trans*-splicing within the conserved sequences of the 5' CS region of DENV. These introns cleave either single stranded or homologously paired double stranded RNA at defined uracils and covalently join a 3' exon tag to the end of the cleavage product. We evaluated these introns for activity in both transfected and transformed cell cultures to determine their effectiveness in targeting DENV sequences. Two of these introns, designed 9v1 and 96v4 gave the greatest number of *trans*-splice product compared to the other Anti-DENV Group I *trans*-splicing introns (αDENV-GrpI) in each respective series, as judged by luciferase assays. The success of this approach against both subgenomic DENV sequences and infecting DENV genomes provides a potent anti-viral strategy that should prove useful against this important disease.

## Results

### Analysis of highly conserved elements in the Dengue genome

**Figure 1:**
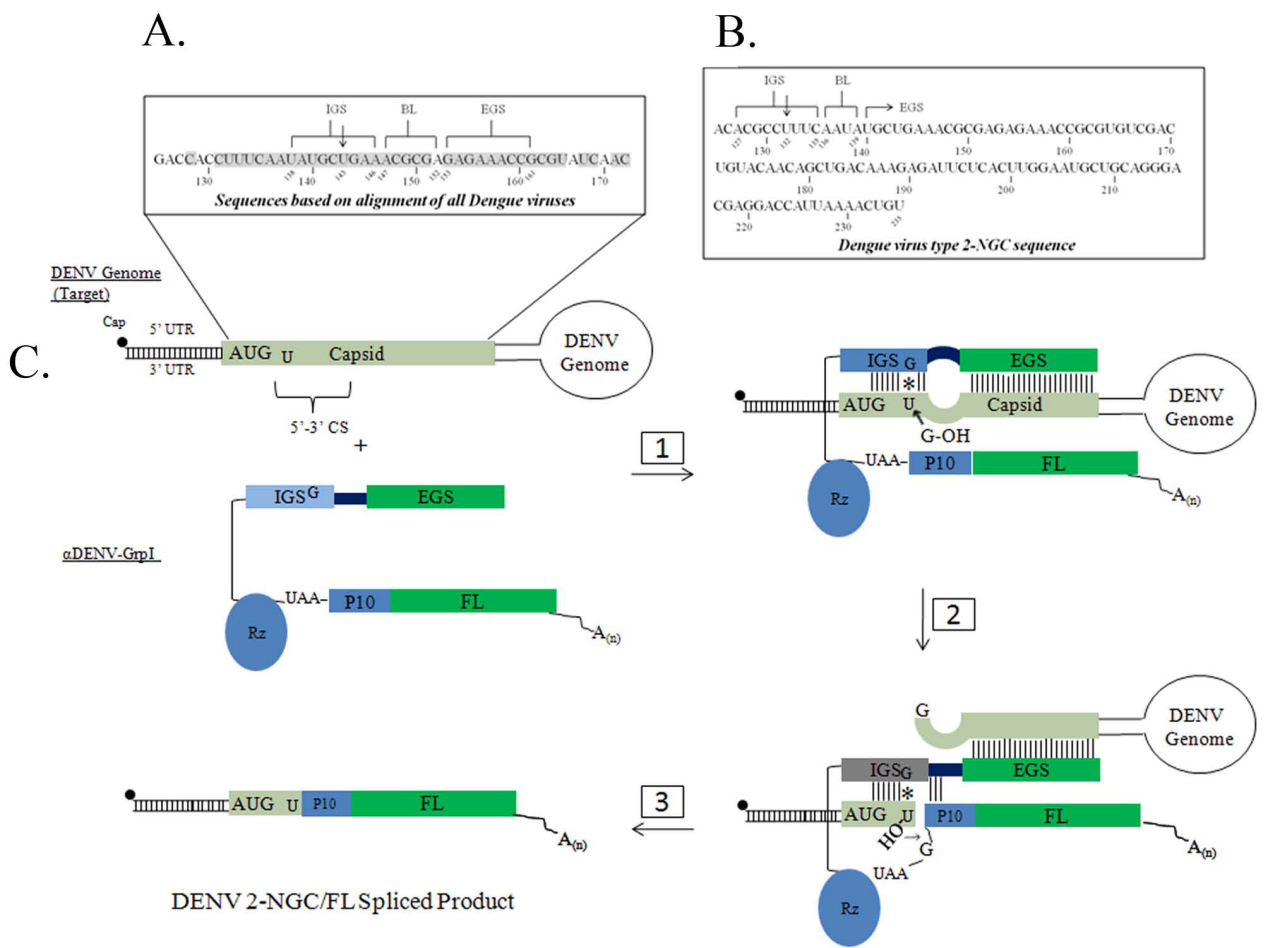
**αDENV-GrpI targeting and *trans*-splicing of DENV**. Schematic diagrams showing DENV sequences targeted by 9 series (A) and 96 series (B) αDENV-GrpIs (shown as blow-ups), and the *trans*-splicing reaction mediated by these introns (C) as previously described for other *trans*-splicing ribozymes (5). A) A region of the DENV genome targeted by the 9 series intron is conserved among all serotypes based on Clustal X DENV genome alignment of all 98 DENV sequences deposited at GenBank. All accession numbers are listed in Methods. Shaded areas indicate 100% conserved bases. The arrows designate U residues targeted for cleavage. The residues contributing to structural elements of the intron RNA-target RNA complexes in the different 9 series introns are as follows: Internal Guide Sequence (IGS) = U138 to A146, Bulge Loop (BL) = A147 to A 152, and External Guide Sequence (EGS) = G153 to G161 as indicated on. Uracil 143 is targeted for cleavage. The single adenine at base 152 is not fully conserved and has therefore been engineered into LB, which is not required to base pair with the target for efficient splicing. B) Primary sequence of the DENV 2-NGC genome depicting the residues contributing to structural elements of the intron RNA-target RNA complexes in the different 96 series intron structures: IGS = A127 to C135, LB = A136 to A 139, and EGS = U140 to U235. The uracil at position 132 is targeted for cleavage. C) The αDENV-GrpI- mediated *trans*-splicing reaction. The DENV target sequence is represented as the upper schematic diagram with the *trans*-splicing ribozymes shown below. See text for description. Figure not drawn to scale.

All nucleotide position designations used throughout the study are relative to the published DENV- 2 New Guinea strain C genome (DENV-2 NGC; GenBank Accession: M29095). We began by aligning all 98 DENV genomes and genome fragments from the four different serotypes that were present in GenBank using the ClustalX program. While overall similarity was highest within a given serotype, the alignment showed a significant conserved region between 131 and 164 nt having only one variable base at position 152 nt (Figure 1a). This sequence was wholly contained within the Capsid protein gene, and overlapped with the 5'CS identified as essential for replication [23,25].

### Design of anti-DENV Group I *trans*-splicing introns (αDENV-GrpIs) targeting conserved DENV sequences

**Figure 2:**
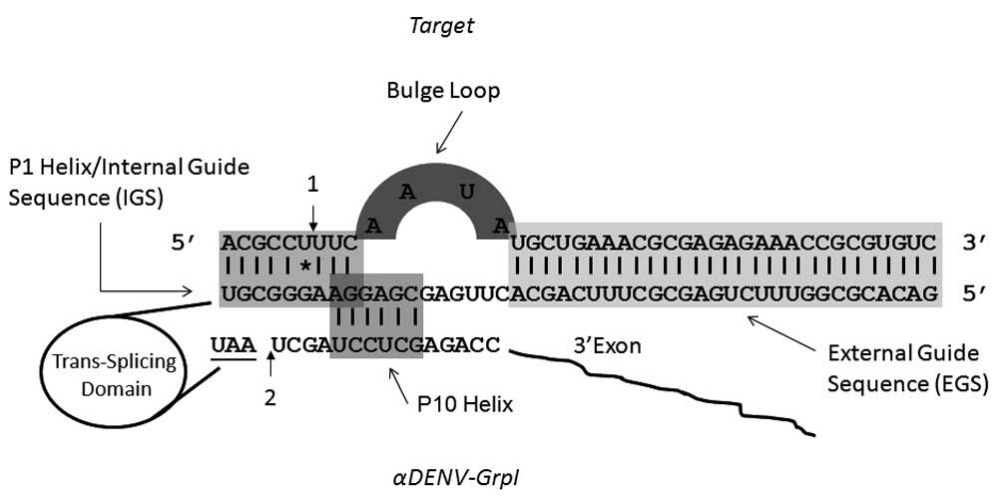
**Anatomy of *trans*-splicing group I introns**. The IGS forms the P1 helix with the target to bring it into proximity with the GNP 3'-OH. The EGS is an extra stretch of antisense RNA that improves the efficiency and specificity of intron targeting the desired viral sequence. The formation of the P10 helix brings the newly cleaved 3'-OH of the uracil into proximity with the 3' exon, allowing covalent splicing. P1 and P10 overlap by 2 bp IGS upon targeting of the DENV-2 genome. A UAA stop codon was inserted in the *trans*-splicing domain of each intron immediately upstream of the UCG splice site to prevent inadvertent translation of the 3' exon until formation of the DENV 2-FL spliced product. The sites of *trans*-esterification on the phosphodiester backbone (1 and 2) are marked with black arrows.

The Group I intron requires an accessible uracil nucleotide downstream of which the target sequence is cleaved. In a *trans*-splicing reaction, two separate segments of the intron are utilized to specify the RNA sequence the ribozyme targets. The internal guide sequence (IGS), a part of the P1 helix, and external guide sequence (EGS) are each complementary to the target RNA sequence (Figure 1c step 1 and Figure 2). The IGS is limited in size to 9 base pairs near the reactive uracil while the EGS can be of any length and forms a transient helix with the target RNA sequence downstream of the reactive uracil [20]. Biochemical analysis has shown that the activity of Group I introns can be enhanced through 5'- and 3'-splice junction base pairing [26].

T*rans*-splicing group I introns promote the joining of a target sequence to a 3' exon through two successive yet independent trans-splicing reactions (Figure 1c; [6]). The IGS and EGS of the αDENV-GrpI form Watson-Crick base pairs with the DENV 2-NGC target RNAs. Unintentional expression of the 3' exon, FL, is prevented by an exogenously inserted UAA stop codon immediately upstream of the UCG splice site. An initial transesterification reaction results in cleavage of the target RNA in a guanoisine dependent manner with conformational change of the EGS. Displacement of the distal portion of the P1helix by sequences upstream of the 3' exon forms the P10 helix, allowing a second transesterification reaction to take place. This results in ligation of the DENV 2 NGC genome to the FL 3'exon, promoting firefly expression from the DENV capsid AUG.

While most strategies for identifying optimal IGS sequences utilize a randomized library, called a GN5 library, to locate the most accessible uracil within a given target sequence [27,28], our approach for targeting a specific segment of the DENV genome within the DENV 5' conserved region limited our choices of uracils, and the GN5 library approach was not an option. We therefore employed a more direct approach for our analyses.

Anti-DENV Group I *trans*-splicing introns (αDENV-GrpIs) were designed to target two different uracil bases within the identified conserved region. The first set of introns targeted uracil 143 (U143) and were designed to effectively *trans*-splice all known DENV sequences (Figure 1a). All of these introns included a 9 nt antisense External Guide Sequence (EGS) targeting downstream sequences that are also conserved among all DENV genomes to improve the targeting capability of the intron and to minimize potential off-target splicing interactions. A single variable base at nt 152 is positioned within a non-homologous bulge loop structure that separates the IGS and EGS, and therefore does not influence the targeting of the intron (Figure 2). This bulge loop structure allows the formation of the P10 helix which increases the catalytic efficiency of the intron [29].

Intron 9v1 was made with a 9 base P1 helix, and a 9 base EGS (Additional file 1). Excluding the wobble base at position U143 which is required for proper cleavage [30-33], 17 bases of this intron interact directly with the intended target sequence.

A second set of αDENV-GrpIs were constructed with an extended 96 base antisense EGS that was engineered to specifically bind to the DENV-2 NGC (Figure 1b). Each version of this series shared the same EGS and P1 helix, and targeted the same uracil, U132, but differed in their P10 helix. The first version of the 96 series, 96v1, was made with a 6 base pair P10 helix that had no wobble base, and a standard P1 helix of 9 bases, inclusive of the required wobble base (Additional file 1). 96v3 is similar to 96v1 in all respects except the trimming of 3 nucleotides between the P10 helix region and the catalytic core. Finally, version 4 of the 96 series (96v4) incorporated a wobble base pairing downstream of the 3' exon splice-site. This alteration in the P10 helix has been used in published experiments with other group I introns [9]. The 96 series of introns target a different uracil than 9 due to the larger stretch of 100% conserved sequence available for base pairing in the Dengue 2 alignment, thus the reason for the differences in the nucleotide content of the IGS and nucleotide content and length of the EGS sequences.

### Assessing αDENV-GrpI activity by firefly luciferase assay in S2 cells

**Figure 3:**
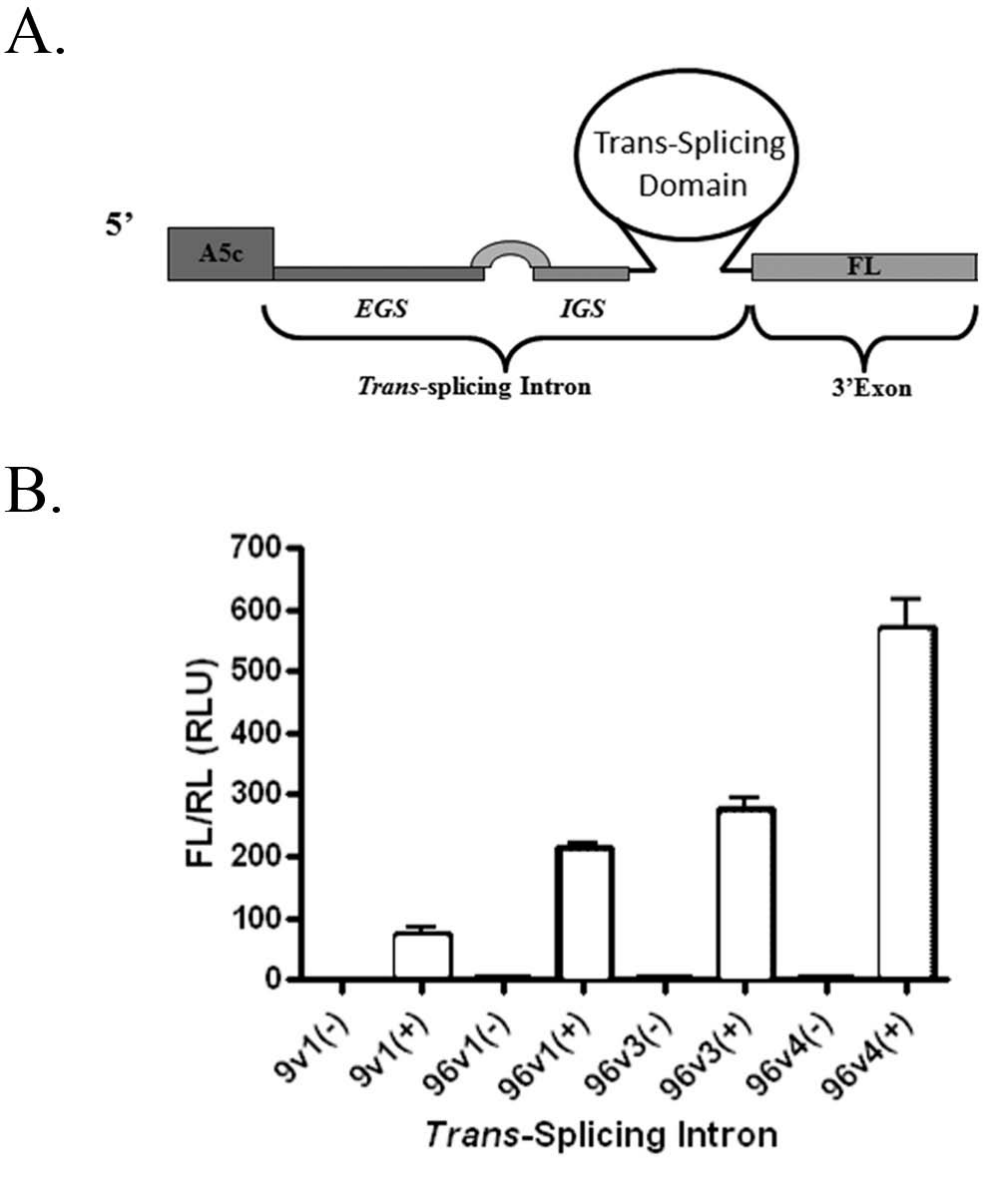
**Quantitative luciferase analysis of αDENV-GrpIs splicing efficiency**. A) Schematic diagram of the αDENV-GrpI-FL constructs used for this analysis. A5c = *Drosophila melanogaster *actin 5c promoter, FL = firefly luciferase. B) *Drosophila *S2 cells were co-transfected with 3 μg αDENV-GrpIs, 0.5 μg of the IRL expression plasmid, and 1 μg of either double-stranded DENV-2 target (+) or control plasmid, pUC57 (-) (see Methods). Following cell lysis, samples were processed using the Dual Luciferase System (Promega) according to the manufacturer's direction. The error bars represent standard deviations of three independent experiments.

Since each αDENV-GrpI was constructed with a 3' firefly luciferase (FL) ORF (Figure 3a), we were able to utilize a standard dual luciferase assay to assess the ability of each αDENV-GrpI to target the 5'CS region and form a DENV2-FL splice product. The formation of this functional splice product can be used to quantitatively gauge the ability of our anti-DENV group I introns to reprogram a target sequence either in the form of a double-stranded fold back mimic, or in the context of DENV infection.

Drosophila S2 cells were co-transfected with αDENV-GrpI expression plasmids possessing FL as the 3' exon, dsDENV-2 substrate expression plasmids, and a *Renilla *luciferase expression plasmid, pA5c-IRL, to normalize the readings (Figure 3b). As a negative control, cells were co-transfected with the pUC57 empty vector as a substitute for the substrate, at the same concentration as the dsDENV-2 concentration used in the experimentals. S2 cells were harvested 48 hours post-transfection, processed and analyzed as described in Methods. All introns demonstrated firefly luciferase activity, and therefore successful targeting of the pA5c-D2-EYFP-D2 construct. αDENV-GrpI 96v4 produced the greatest amount of luciferase activity, and therefore the greatest amount of *trans*-spliced product, of all the 96 intron series studied (Figure 3b). The αDENV-GrpI as9v1, designed to target all DENV serotypes, also produced significant FL activity verifying its ability to attack our DENV mimic in these cells.

### Engineering and assessment of bicistronic αDENV-GrpI 9v1 and 96v4 intron constructs in *Drosophila *S2 cells

**Figure 4:**
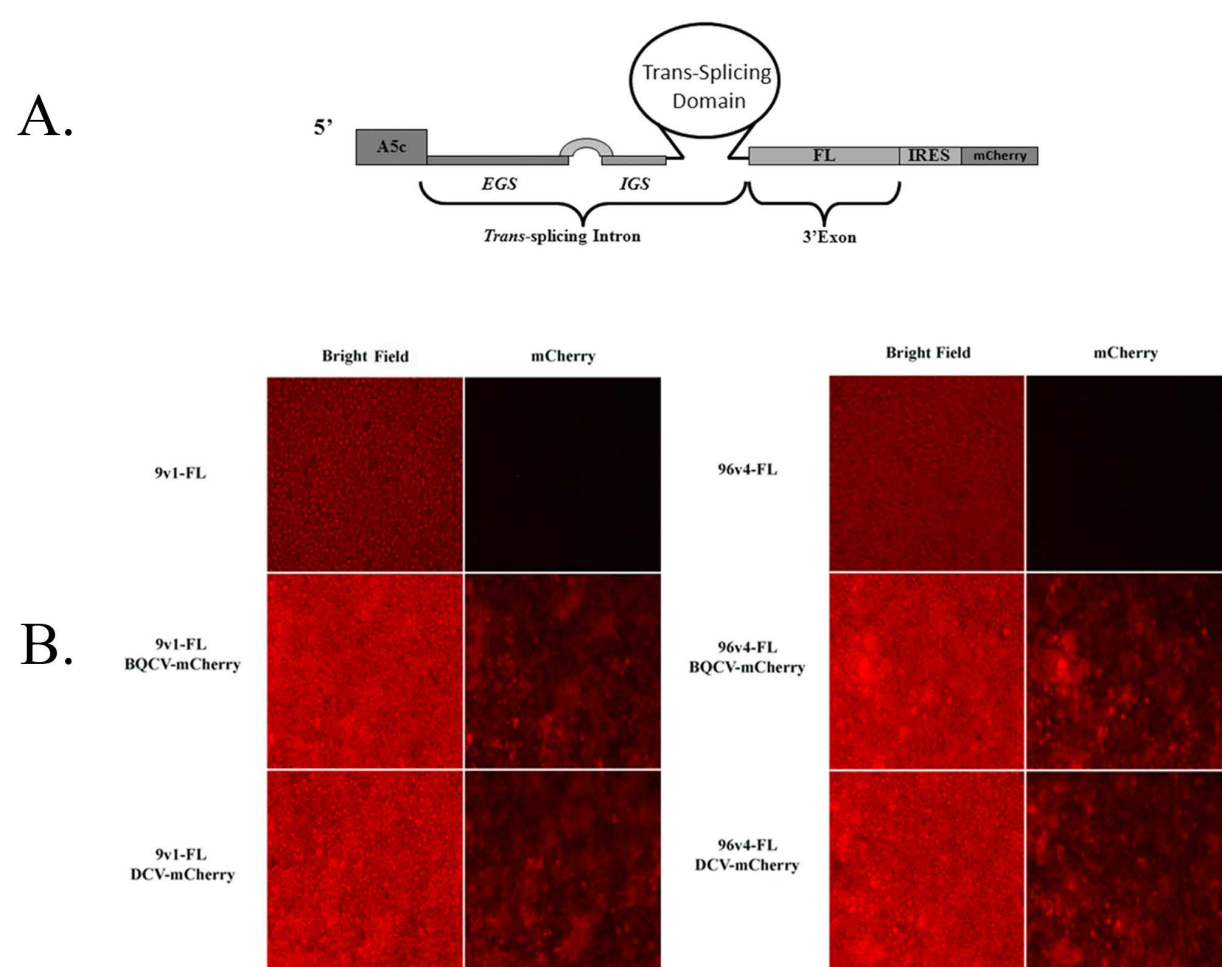
**Engineering and fluorescence microscopy of αDENV-GrpIs in a Bicistronic Plasmid**. A) Each of the *trans*-splicing αDENV-GrpI was tagged downstream of the firefly (FL) 3' exon with the mCherry fluorescent marker gene expressed from an IRES sequence of either the Black Queen Cell Virus (BQCV) or Drosophila C Virus (DCV). Expression of these constructs was driven by the *Drosophila melanogaster *Actin 5c promoter. This bi-cistronic configuration allowed monitoring for the presence and expression of the αDENV-GrpI constructs within cell cultures. A5c = *Drosophila melanogaster *actin 5c promoter, IRES = DCV or BQCV Internal ribosome entry site. B) Expression of mCherry was verified for each construct by transfecting 1 ug of plasmid DNA into C6/36 cells and examining at 48 hours post transfection. Photographs were taken under 40× magnification.

Since αDENV-GrpIs 9v1 and 96v4 were determined to be the best candidate introns of each series by the *in vitro *assay we assessed the activities of these introns in transfected cell culture assays. Each of the introns was tagged downstream of the 3' exon with the mCherry fluorescent marker gene expressed from an IRES sequence of either the Black queen cell virus (BQCV) or Drosophila C virus (DCV) (Figure 4a). These Dicistrovirus IRES sequences were previously determined to yield the highest levels of expression in *Ae aegypti *mosquito and *D. melanogaster *S2 cells [34]. This bi-cistronic configuration allowed monitoring for the presence and expression of the αDENV-GrpI constructs within cell cultures. As expected, IRES-mediated expression of the mCherry fluorescent marker occurred upon transfection of these bi-cistronic constructs in S2 cells (Figure 4b.).

**Figure 5:**
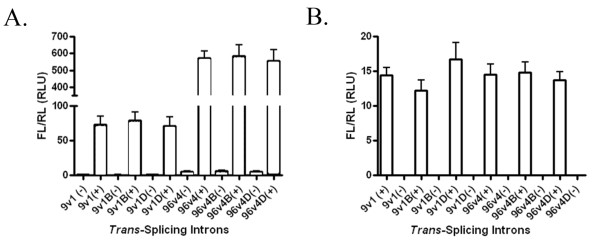
**Assessment of αDENV-GrpI bicistronic constructs activities using luciferase assays**. A) *Drosophila *S2 cells were co-transfected with 3 μg of each αDENV-GrpI, 1 μg double-stranded DENV-2 target (+) or control plasmid (-), and 0.5 μg of IRL expression plasmids. Following cell lysis, samples were processed and assayed for luciferase activity (see Methods). The error bars represent standard deviations of three independent experiments. B) Aag2 cells co-transfected with αDENV-GrpI and pA5c-IRL expression plasmids were challenged with DENV-2 NGC at an MOI of 0.01 24 h post transfection (+). Control cells were transfected with an empty pUC57 plasmid (-) and challenged with virus in the same manner. The error bars represent standard deviations of three independent experiments. B = BQCV IRES. D = DCV IRES. RL = *Renilla *luciferase. RLU = Relative Luciferase Units.

We examined the potential influence addition of the IRES-mCherry configuration had on αDENV-GrpI activity by performing dual luciferase assays (see Methods) with our bicistronic αDENV-GrpIs 9v1 and 96v4 intron constructs in transient transfected cell culture (Figure 5a). *D. melanogaster *S2 cells were transiently transfected with αDENV GrpI 9v1 or 96v4 either unlinked or linked to an IRES/mCherry driver, and were challenged with a double-stranded fold back construct designed to mimic the 5'-3' CS region of DENV-2 (dsDENV-2; see Methods) in the presence of the pA5c-IRL normalizer. To rule out non-specific targeting of the anti-DENV introns to cellular targets, control cells were transfected with the pUC57 plasmid in lieu of the dsDENV-2 construct.

FL activity was greatest for the 96v4 IRES-mCherry-linked or unlinked constructs in S2 cells (Figure 5a), with no statistical differences in activities among the 96v4 intron constructs. This confirms that addition of the 3' IRES/mCherry configuration does not alter the *trans*-splicing capabilities of the 96v4 intron, since equivalent FL counts were obtained for both 96v4 and 96v4 IRES/mCherry constructs.

Similarly, the overall activities of the 9v1 intron constructs, whether IRES-mCherry linked or unlinked, were statistically similar. However, the overall levels of activation were substantially lower than those detected in cells expressing 96v4 introns, possibly due to the shorter EGS [7] target accessibility leading to a decrease in the production of *trans*-spliced product.

**Figure 6:**
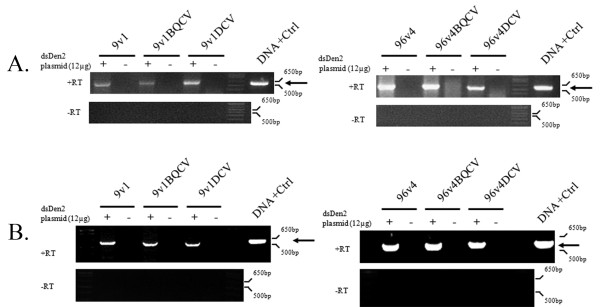
**αDENV-GrpI constructs effectively target DENV-2 NGC**. T-25 flasks containing *D. melanogaster *S2 cells (5 × 10^5^) transiently transfected (A) or transformed (B) with αDENV-GrpI bicistronic constructs, were transfected with double-stranded DENV-2 target (+), or the negative control pUC57 empty vector (-). Resulting RNAs were analyzed by RT-PCR in the presence (+Rt) or absence (-Rt) of reverse transcriptase to insure that observed amplified products were derived from RNA. A PCR amplification product derived from a constructed spliced sequence control (Methods) is provided as size standard for each gel (DNA+Ctrl). Arrows indicate the predicted size of the principle splice products resulting from intron activity. The identity of splice product was confirmed by sequencing.

αDENV-GrpIs 9v1 or 96v4, IRES-mCherry linked or unlinked, were either transiently or stably expressed in S2 cells, and analyzed by RT-PCR 72 hours post-transfection with the dsDENV-2 target plasmid using heterologous primers (see Methods). Splice product bands were excised, gel purified, and sequenced to confirm their identity. The specific DENV-FL splice product was detected by RT-PCR in transfections with both αDENV-GrpI 9v1 and 96v4 in S2 cells as evidenced by the presence of a 580 bp band, no splice product was detected in the absence of the target dsDENV-2 expression plasmid (Figure 6a and 6b).

### αDENV-GrpIs effectively target DENV-2 NGC in mosquito cells

The effectiveness of our αDENV-GrpI introns to target infecting DENV genomes was assessed by FL assays following DENV-2 challenge of *Ae. aegypti *Aag2 cells transiently transfected with αDENV-GrpI introns (Figure 5b). αDENV-GrpI and pA5c-IRL expression plasmids were co-transfected into Aag2 cells, and were challenged with DENV-2 NGC at an MOI of 0.01 24 h post transfection. Control cells were transfected with an empty pUC57 plasmid, in place of the plasmids and challenged with virus in the same manner. Each of the αDENV-GrpIs displayed levels of FL activity indicating successful splicing against the infecting DENV. In this case FL activity was only slightly greater for the 96v4 IRES-mCherry-linked or unlinked constructs in Aag2 cells, with no statistical differences in activities among the 96v4 intron constructs. Similarly, the overall activities of the 9v1 intron constructs, whether IRES-mCherry linked or unlinked, were statistically similar. As seen in the previous assays, the overall levels of activation were somewhat lower than those detected in cells expressing 96v4 introns most likely due to the shorter EGS [7] or target accessibility. This confirmed the activity of our αDENV-GrpIs against actual infecting virus, and demonstrated that addition of the 3' IRES/mCherry configuration does not appear to alter the ability of the αDENV-GrpIs tested to target DENV genomes in cells. The overall levels of luciferase activity were lower in these virally infected mosquito cells than those observed in S2 cells transfected with a plasmid construct expressing an artificial target sequence. This may be due to the role viral infection plays in host cell RNA and protein expression, or may be due to potential basic differences in nascent RNA and protein expression between these two cell lines.

**Figure 7:**
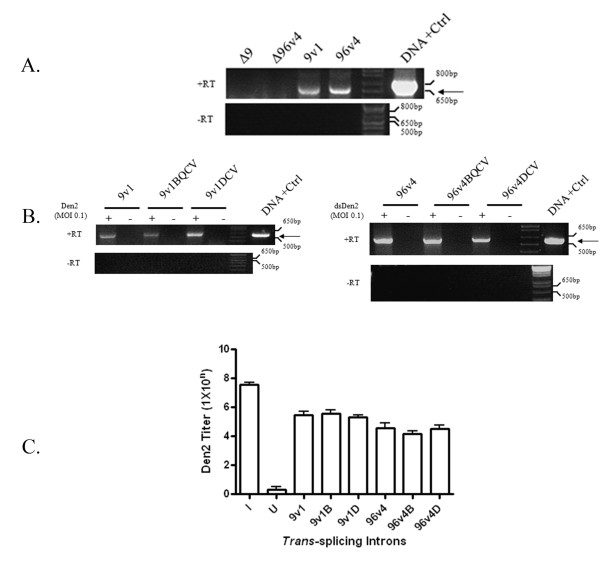
**αDENV-GrpI -FL constructs effectively target and suppress DENV-2 in mosquito cells**. A) Confirmation of representative αDENV-GrpI trans-splicing activities. *Ae. Albopictus *C6/36 cells were transiently transfected with *trans*-splicing αDENV-GrpI bicistronic vector constructs. Resulting RNAs were analyzed by RT-PCR in the presence (+Rt) or absence (-Rt) of reverse transcriptase to insure observed amplified products were derived from RNA. A PCR amplification product derived from a constructed spliced sequence control (Methods) is provided as a size standard for each gel (DNA+Ctrl). Δ9 and Δ96 refer to Pabc5 deletion mutations located in the *trans*-splicing domain of the group I intron. These deletions are designed to knock out function providing an adequate negative control [52]. Arrows indicate the predicted size of the principle splice products resulting from intron activity. The identity of spliced product was confirmed by sequencing. B) C6/36 cells were transformed with *trans*-splicing αDENV-GrpI bicistronic vector constructs and maintained under 10 mg/ml hygromycin selection for more than 30 doublings. Transformed cells were washed twice in serum free media at 15 hours post plating, infected with DENV-2 NGC (MOI = 0.1). RNAs were analyzed by RT-PCR as described in A). Arrows indicate the predicted size of the principle splice products. C) *Ae. albopictus *C6/36 cells were transformed with group I intron vector constructs and maintained under hygromycin selection. Transformed cells were washed three times and challenged with DENV 2-NGC (MOI 0.01). Infections were allowed to proceed for 4 days, supernatants were collected, and viral titers were determined by TCID_50_-IFA as described in Methods.

Transient transfection of 9v1, 96v4 and inactive ribozymes Δ9 and Δ96 was performed in C6/36 cells followed by RT-PCR analysis to confirm the detection of splice product (Figure 7a). No splice product was observed in the presence of the inactive ribozymes Δ9 and Δ96 showing that the splice product detected is due to the *trans*-splicing activities of the αDENV-GrpIs.

Since αDENV- GrpIs 9v1 and 96v4 were determined to effectively target DENV genomes in transiently transfected cells, we assessed the activities of these introns in transformed mosquito cell culture assays (Figure 7b). To produce *Ae. albopictus *C6/36 cells transformed with each bicistronic αDENV-GrpI intron construct, cells were co-transfected with each αDENV-GrpI construct and a plasmid possessing the hygromycin resistance gene. Transfection media was replaced with selective media at 48 hours post transfection. Cells were then passaged several times per week in selection media. The concentration of hygromycin used was increased with each passage until a final concentration of 10 mg/ml was reached. mCherry fluorescence and RT-PCR were used to confirm expression of the introns in the transformed cultures.

αDENV-GrpIs 9v1 and 96v4 linked to either the BQCV or DCV IRES elements expressing mCherry were stably expressed in *Ae. albopictus *C6/36 cells and challenged with DENV-2 NGC at an MOI of 0.1 at 24 h post transfection. Control cells were transfected with an empty pUC57 plasmid and challenged with virus in the same way (Figure 7b).

Cells were processed and analyzed by RT-PCR 4 days post-infection with heterologous primers to detect the DENV-FL splice product, and identified bands were excised, gel purified, and sequenced to confirm their identity. DENV-2-FL splice product was detected in C6/36 cells when introns were expressed in a transformed cell manner, and whether the intron was linked with either IRES-mCherry configuration (Figure 7b). No control DENV-2-FL splice product was detected by RT-PCR in cells transfected with the pUC57 control vector. Significantly, these results also show that the 9v1 intron, designed to be active against all forms of Dengue virus, is capable of effectively targeting the DENV 2-NGC genome in a sequence specific manner.

### Expression of αDENV-GrpIs 9v1 and 96v4 in mosquito cells leads to suppression of DENV-2 NGC

The final step in our analysis of these αDENV-GrpI constructs was to determine their ability to suppress overall infectious DENV-2 NGC production in cell culture using tissue culture infectious dose immunofluorescence antibody (TCID_50_-IFA) assays (Figure 7c; [4]). αDENV-GrpI-FL constructs were stably expressed in C6/36 cells, challenged with DENV-2 NGC, and assayed as described in Methods. αDENV-GrpIC6/36 cell lines 9v1 and 96 v4 displayed vast reductions in viral titer, up to 3 log, when compared to the infection control (I). Suppression of virus replication is evident regardless of whether the intron expressed in the cells was the 96v4 *trans*-splicing intron, engineered to specifically target DENV-2, or the 9v1 *trans*-splicing intron, which was designed to target all Dengue virus serotypes. This anti-viral effect was independent of the IRES-mCherry configuration used in the anti-DENV constructs. Though the 96v4 intron appeared to suppress DENV-2 NGC replication to a greater extent than 9v1, a direct comparison of activities cannot be considered valid since αDENV-GrpIs 9v1 and 96v4 target different uracils (4).

## Discussion

Like other Flaviviruses, DENV enter the cell by receptor mediated endocytosis ([35,36]; RME)). Following acidification of the endosome and membrane fusion the 9.6 kb positive-sensed DENV genome is released into the cytoplasm where replication begins. This is an ideal place for a *trans*-splicing ribozyme to attack the DENV genome. One strategy currently under development to directly attack the Dengue genome is the use of the RNAi response in mosquitoes [37-42]. It is logical to assume that the wild-type RNAi response mounted by the mosquito itself in reaction to an infection is not strong enough to protect against the spread of the virus, or Dengue would cease to be a concern. However, pre-priming mosquito cells for RNAi protection against Dengue through the expression of Dengue-specific dsRNA before any infection occurs is an effective approach, severely hindering replication of the virus in some cases [39]. As efficient as it is, this tactic suffers from the same drawbacks as the vaccine: escape mutants. The error rate of the Dengue RNA polymerase suggests that, on average, one random mutation arises for every replication event. Previous work has shown limited mismatching of the RISC complex RNA to its target is tolerated to a degree [11]. In contrast, the ability of an induced RNAi response to discriminate between different alleles of the same gene varying only by a single nucleotide has also been observed [43]. Whether or not a particular mutation within the targeted region confers resistance appears to depend on the location of the altered base as well as the nature of the alteration itself. Selective pressure within the cells of a mosquito may initially silence a Dengue infection, but eventually that pressure would serve to promote the replication of virus genomes carrying mutations conferring resistance to the RISC complex nuclease activity. Such mutants would be transmitted by the insect and spread throughout a population even in the presence of the protective measures granted by RNAi, eventually rendering the specific sequence utilized in priming a mosquito for an RNAi response useless in the face of the escape mutant strain of Dengue.

The experiments presented here are not conducted in a way that one can assess if the Grp-1 approach is superior or inferior to siRNA/shRNAs. siRNA does not carry out splicing, and our research was meant to establish that we had successfully designed GrpI introns that do target and splice conserved sequences. Further, an abundance of siRNA work has already been performed with DENV [44,45] and this sequence is not among those identified as useful for targeting because the length of conservation for this sequence among all DENV is smaller than required for an siRNA response.

Group I *trans*-splicing introns have a demonstrated potential for targeting RNA virus genomes in infected cells [20,21]. In this report we demonstrate the feasibility of using αDENV-GrpIs to catalyze *trans*-splicing of the 5' conserved region of the DENV family genomes. In designing Group I intron splicing approaches most studies employ a GN5 library scan to map those uracils most accessible to *trans*-splicing in an otherwise highly invariant sequence [28]. The highly mutable nature of the Dengue genome precludes this approach, as any uracils identified may or may not be present in other serotypes or even other strains of the same serotype.

In addition to the questionable presence of the uracil, the immediate neighboring sequence must also be conserved to facilitate targeting through base pairing interactions. We determined our optimal Group I intron target following alignment of 98 instances of DENV from GenBank which identified one conserved region that appeared to satisfy the requirements for *trans*-splicing within the DENV genome. This region is positioned within the capsid coding sequence at nucleotides C131 to G151 [23,25], and contains a number of possible uracil targets for the *trans*-splicing reaction. This region is a part of a double-stranded 5'-3'CS domain that forms as a result of complementary base pairing between the proximal ends of the 5' and 3' UTRs during DENV replication [23]. The formation of the 5-3'CS domain has been shown to be essential for DENV replication [25]. We designed anti-DENV Group I *trans*-splicing introns (αDENV-GrpIs) to target two of these uracil bases within the identified conserved region, U143 and U132.

Intron 9v1, which has a 9 base P1 helix, and a 9 base EGS (Additional file 1), is designed to effectively *trans*-splice all known DENV sequences. This intron demonstrated an ability to cleave at U143 and effectively *trans*-splice an infecting DENV 2 NGC genome either upon transfection of Aag2 cells or as a constitutively expressed RNA in transformed C6/36 cells.

A separate set of αDENV-GrpIs were constructed with an extended 96 base antisense EGS that was engineered to target the DENV-2 NGC (Figure 1b). Each version of this series shares the same EGS and P1 helix, and targets U132, but differs in their P10 helix. 96v1 has a 6 base pair P10 helix with no wobble base, and a standard P1 helix including the required wobble base (Additional file 1). 96v3 differs from 96v1 in trimming of 3 nucleotides between the P10 helix and the catalytic core, while 96v4 incorporates a wobble base pairing downstream of the 3' exon splice-site.

Each αDENV-GrpI was constructed with a 3' firefly luciferase (FL) ORF that permitted quantitative assessment of splicing activity. Co-transfection assays for FL activity were performed in S2 or Aag2 cells using the fold back DENV-2 mimic plasmid and either BQCV or DCV IRES/mCherry-linked αDENV-GrpI 9v1 or 96v4expression plasmids introns. Although all introns assayed exhibited firefly luciferase activity, αDENV-GrpI as96v4 displayed the greatest amount of luciferase activity, and therefore the greatest amount of *trans*-spliced product. This is likely due to both the relative activity of the intron configuration as well as an increased efficiency of targeting as a result of the extended EGS. The αDENV-GrpI as9v1, designed to target all DENV serotypes, also displayed a significant ability to successfully splice our DENV mimic in these cells, but its reduced level of FL activity reflects a somewhat reduced efficiency of targeting relative to αDENV-GrpI as96v4. This is likely due to the presence of a shorter EGS, as this has been previously shown to decrease the ability of a *trans*-splicing intron to attack a target sequence [7]. Alternatively, the relative effectiveness of cleavage for U143 targeted by the 9v1 intron may be less than that for U132 targeted by the 96v4 introns. Nonetheless, this intron was still quite effective in targeting and splicing the DENV sequence.

Similar results were obtained when Aag2 cells transfected with these αDENV GrpI constructs were challenged by infection with DENV-2 NGC. These results validated the potential of our αDENV-GrpI intron approach as an effective means of suppressing DENV infection of mosquito cells and tissues.

We also observed that addition of a 3' IRES/mCherry configuration, whether incorporating the BQCV IRES or the DCV IRES, does not appear to alter the *trans*-splicing capabilities of either intron, Because the IRES allows expression of the mCherry fluorescence marker in the unspliced intron, this provides a convenient independent marker for determining the relative efficiency of expression of the introns following transfection.

The potential of these intron constructs to function in transformed mosquito tissues was confirmed by demonstrating their activity against infectious DENV-2 NGC in transformed C6/36 cells expressing the bicistronic αDENV-GrpIs 9v1 or 96v4, either linked with the BQCV or DCV IRES driven mCherry, or lacking an IRES mCherry linkage (Figure 7). RT-PCR amplified DENV-2-FL splice product confirms the ability of both αDENV GrpI 9v1 and 96v4 constructs in targeting DENV-2 NGC. A decreased band intensity of the DENV-2-FL splice product was obtained with αDENV-GrpI 9v1 compared to that resulting from αDENV-GrpI 96v4 activity, and may once again be due to differences in the lengths EGS of these two introns since EGS length is a determining factor for GrpI *trans*-splicing efficiency [7].

Further validation of these introns as potent tools to combat DENV is evidenced by TCID_50_-IFA analyses that test suppression of overall infectious virus production (Figure 7). A 2 log (for 9v1) to 3 log (for 96v4) was observed demonstrating these αDENV-GrpIs do suppress DENV 2-NGC infection of the transformed cells, and the αDENV-GrpI 9v1, designed to target all four Dengue serotypes, has the ability to suppress the replication of all serotypes of this virus.

## Conclusions

The results presented here show that the 9v1 intron, designed to be active against all forms of Dengue virus, is capable of effectively targeting the DENV 2-NGC genome in a sequence specific manner, while suppressing virus production. These novel αDENV-GrpIs provide an attractive alternative to other RNA based approaches for the transgenic suppression of DENV in transformed mosquito cells and tissues.

## Methods

### Alignment of all DENV genomes

DENV sequence data was obtained from the national center of biotechnology information (NCBI). Sequences representative of all four serotypes of Dengue were aligned using ClustalX [46]. The aligned sequences comprise the following GenBank GenInfo identifiers: 12018173, 12018169, 12018171, 12659201, 2909798, 2909788, 2909786, 2909796, 6841603, 6841595, 6841605, 6841591, 6841601, 6841597, 6841593, 6841599, 6841587, 6841585, 6841589, 1000740, 1000738, 2909784, 1000736, 4926937, 4926935, 4926927, 4926929, 4926931, 2909794, 2909792, 1000742, 4926933, 2155257, 2723944, 323447, 6581076, 6581078, 2723942, 323449, 323650, 18644123, 1864412, 11119731, 19744844, 18644125, 18644127, 18643733, 4337012, 13386495, 1881708, 19071809, 13926152, 9280544, 14585842, 4926947, 4926939, 323654, 4926945, 4926943, 7329983, 7329981, 13540386, 14328931, 14485523, 323660, 17129645, 22901065, 22901063, 22901061, 1854040, 1854038, 1854036, 17129647, 24417519, 24417517, 24417515, 27656962, 24417513, 19071807, 14195698, 8927332, 14328929, 12711599, 323468, 25992053, 25992047, 25992041, 25992029, 25992025, 25992055, 25992033, 19071811, 25992043, 25992039, 25992037, 25992051, 25992031, and 25992057.

### Cells, Virus and Antibody

The *Drosophila melanogaster *In S2 cells, obtained from ATCC, were maintained in Schneider's Modified Drosophila media (Invitrogen/Gibco) 10% FBS (Atlanta Biologicals), penicillin G (100 U/ml; Invitrogen/Gibco) and streptomycin (100 μU/ml; Invitrogen/Gibco). Aag2 *Aedes aegypti *mosquito cells (a kind gift from Dr. Ken Olson, Colorado State University, Fort Collins, CO) were maintained in Schneider's Modified Drosophila media (Lonza Group Ltd., Walkersville, MD, USA) supplemented with 10% FBS, 2 mM glutamine, penicillin G and streptomycin as described for the S2 cells used in this study. Both cell types were maintained in a 28°C/5% CO_2 _environment.

The Dengue 2 prototype virus New Guinea C strain (DENV2-NGC; a kind gift from Dr. Stephen Higgs, UTMB, Galveston, TX) was used in this study. Viral stocks were prepared as follows. Aag2 cells were infected with DENV 2-NGC. At 7 days post-infection, cells were scraped and freeze-thawed for three (3) cycles. Cell debris was removed by spinning cell suspensions at 10000 RPM for 30 min, and aliquots of 100 ul were stored at -80°C until used, one aliquot was used for determining the TCID _50 _of the stock. This virus stock (TCID _50 _= 10 ^7^) was used in subsequent experiments.

### Design and assembly of anti-Dengue virus group I intron constructs

All primer sequences are illustrated in Additional file 2. All PCR products described here were band isolated using either the QIAquick Gel Extraction kit (Qiagen), or the Wizard SV Gel and PCR Cleanup Kit (Promega) and used as templates for a second round of PCR with the same primers used for the initial PCR reactions and Platinum *Pfx *high-fidelity DNA polymerase according to the manufacturer's protocols in order to minimize the amount of contaminating circular plasmid. Following band isolation all PCR products were digested with restriction endonucleases, obtained from New England Biolabs (NEB). All vectors were Antarctic phosphatase treated (NEB) prior to ligation. The final constructs produced were sequenced and restriction digested to verify plasmid integrity and presence of the inserts.

#### pA5c backbone

The αDENV-GrpI were cloned into an expression vector under the control of the distal *Drosophila melanogaster *actin5c. The actin5c promoter was PCR amplified from the plasmid pHermes [Actin5c:EGFP] [47].' Following band isolation the PCR product and the vector pBlueScriptII SK+ (Stratagene, La Jolla, CA) were band isolated and digested with the restriction enzymes *Acc*65I and *Not*I, and ligated using T4 DNA ligase (NEB) to give the plasmid pBlueScriptII SK+ Actin5c (pBSII-A5c). The SV40 late transcription terminator and polyadenylation signal was amplified from the plasmid pMT/V5-HisA (Invitrogen). The SV40 PCR product and pBSII-A5c were digested with *Not*I and *Sac*I (NEB). The plasmid and insert were ligated together with T4 DNA ligase to yield the vector pA5c, and served as the backbone for the rest of the plasmids produced unless otherwise noted.

#### pA5c-FL

The firefly luciferase open reading frame was PCR amplified from the vector pGL-Basic (Promega). The PCR product and pA5c vector were a digested with *Xho*I and *Not*I. and ligated to yield pA5c-FL.

#### Group I introns

All introns were derived from the catalytic core of the rRNA *Tetrahymena thermophila *group I intron on the pTT1A3-T7 plasmid (Kind gift of Dr. Thomas R. Cech; [48]). The 9 series αDENV-GrpIs Δ9 and 9v1 as well as the 96 series Δ96 96v1, 96v3, and 96v4, were generated by PCR amplification of the rRNA *Tetrahymena thermophila *group I intron using the primer sets shown in Additional file 2. Following PCR amplification and band isolation the αDENV-GrpIs were digested with MluI and XhoI and inserted into pA5c-FL. The intron designated 9v1 for the length of its antisense region, was amplified, inserted into pA5c-FL, and named pA5c-9v1.

The 96 series introns were all PCR amplified in two steps. An initial PCR template was created by amplification of pTT1A3-T7 with an initial primer set (see 96 series primer set 1 in ST1) and used as a template for a second round of PCR. For this second PCR step each 96 series was amplified with the same forward primer used in the first PCR step, but with different reverse primers for each 96 series intron (see ST1, "96 series primer set 2"). The resulting introns were named pA5c-96v1, pA5c-96v3, and pA5c-96v4.

#### ΔP5abc Introns

To create introns missing the P5abc helix, the catalytic core of the intron was first amplified from pTT1A3-T7 and then inserted into the vector pCR2.1-Topo (Invitrogen) to create pCR2.1-GI. Using opposite facing primers 5' 3' and with the *BsmBI *restriction site at each of their 5' ends, the entire pCR2.1-GI vector containing the intron was amplified, save for the P5abc helix. The resulting PCR product was purified, digested with *BsmBI *and *DpnI *and ligated to itself to form the plasmid pCR2.1-ΔP5GI, which contained the catalytic core of the intron without the P5abc helix.

For purposes of creating control vectors, the intron fragments from plasmid pCR2.1-ΔP5GI were amplified with the same primers as the intron inserts from the 9v1, 96v4 series as described above. The products were band isolated and digested with *Mlu*I and *Xho*I, and inserted into the pA5c-FL using the same restriction sites yield pA5c-Δ9v1 and pA5c-Δ96v1, respectively.

Evaluation of αDENV-GrpIs in S2 cells necessitated the construction of double and single-stranded DENV 2-NGC target constructs. The assembly of these is detailed below.

#### pA5c-EYFP

The DENV 2-NGC target carrier plasmid, pA5c-EYFP, was created by amplification of the EYFP open reading frame from pXL-Bac-EYFP [49]. The PCR product was band isolated and digested with *Mlu*I and *Xho*I, and inserted into the pA5c-FL plasmid using these same sites.

#### pA5c D2EYFP single stranded target plasmid

The yeast shuttle vector pRS424-DENV-2 NGC (a kind gift of Dr. Barry Falgout) was used as the template for PCR amplification of substrate fragments for the production of the single stranded DENV 2-NGC target substrate. PCR products corresponding to nucleotides 85-267 of the DENV 2-NGC genome were digested with the restriction enzymes *Bss*HII and *Mlu*I, and inserted into the *Mlu*I digested pA5c-EYFP.

#### pA5c-D2EYFPD2 double stranded target plasmid

The substrate plasmid bearing the hybridizing sections of the DENV-2 NGC genome at either end of an EYFP open reading frame was made by amplification of the 3' terminus (nt10495-10723) of the DENV-2 NGC genome from pRS424-DENV-2 NGC. The PCR fragment fragment was digested with the restriction enzymes *Xho*I and *Xba*I and inserted into the *Xho*I, *Xba*I digested pA5c-D2EYFP plasmid.

#### pA5c-IRL

The *Renilla *luciferase normalizing plasmid was created by PCR amplification of the chimeric intron and *Renilla *luciferase open reading frame from the plasmid pRL-SV40 (Promega). Following band isolation, PCR fragments were digested with *Xho*I and *Not*I and inserted into the *Xho*I and *Not*I-digested pA5c plasmid.

##### pA5c-DNA+ctrl

The plasmid directing the constitutive expression of an mRNA corresponding to the predicted *trans*-spliced product was made by amplification of the DENV-2 fragment from the pA5c-D2-EYFP plasmid followed by digestion of the PCR fragment and the vector pA5c-FL with *Not*I and *Xho*I, and ligation of these fragments.

#### BQCV and DCV-mCherry bearing αDENV-GrpIs

Production of αDENV-GrpI constructs possessing either the BQCV or DCV intergenic IRES sites linked to an mCherry fluorescent marker was achieved through the insertion of PCR amplified BQCV-mCherry or DCV-mCherry fragments into the pA5c-9v1 and pA5c-96v4 plasmids immediately upstream of the 3' exon, FL (Figure 4a). The BQCV-mCherry and DCV-mCherry fragments were derived unpublished vectors, pA5c-BQCV-mCherry an pA5c-DCV-mCherry. Prior to insertion PCR amplified BQCV-mCherry and DCV-mCherry fragments as well as the 9v1 and 96v4 bearing constructs were digested with *NotI *and *BamHI*. The IRES-linked mCherry PCR fragments were then ligated into the vector constructs.

### Reverse transcription-PCR of DENV 2-firefly luciferase splice products derived from cell culture

The total RNA from Dengue virus infected and uninfected cells was extracted using the Qiashredder and RNeasy Mini kits (QIAGEN Inc., Valencia, CA, USA) in accordance with the manufacturer's instructions and eluted in a final volume of DNAse/RNAse free water to a final volume of 40 μl. The total RNA (5 ug) extracted was treated with 2 U Turbo DNA-free DNAse (Applied Biosystems/Ambion, Inc. Austin, TX USA) to rid samples of any DNA contamination, 30 minutes at 37°C. For DNase inactivation, 0.2 volumes of DNase Inactivation Reagent (Applied Biosystems/Ambion, Inc. Austin, TX USA) was added to each sample tube and incubated at room temperature for 5 minutes, mixing occasionally. One-step RT-PCR was performed using the SuperScript III One-Step RT-PCR kit (Invitrogen) in accordance with the manufacturer's instructions. cDNA synthesis and PCR amplification were performed as follows: 1) cDNA synthesis at 50°C for 45 minutes, 2) 40 cycles: denaturation at 95°C for 2 minutes, annealing at 60°C for 1 min, and extension at 68°C for 2 min, 3) final extension of 68°C for10 minutes. For detection of the DENV-2 NGC- FL spliced product the forward primer 5' TCTGATGAATAAC 3', designed to anneal to DENV2- NGC, and the reverse primer 5' GAACGTGTACATCGACTGAAATCC 3', designed to anneal to FL were used.

### Luciferase assays

Schneider 2 (S2) cells were plated into 9.6 cm^2 ^well in minimal S2 media (Gibco) at a density of 1.0 × 10^6^cells/well. Following the adherence of cells, 3 μg intron, 1 μg double-stranded DENV2 target, and 0.05 μg of IRL expression plasmids were co-transfected into the cells using the Transfectin liposomal transfection reagent (Bio-Rad Laboratories, Hercules, CA) in accordance with the manufacturer's protocol. Transfected cell were incubated at 28°C/5%CO_2 _for 16 hours, washed once in Schneider's media and once in Schneider's media supplemented with 10% FBS and penicillin/strepavidin. These cells were overlaid with Schneider's media supplemented with 10% FBS, 25 μg/mL amphotericin and penicillin/strepavidin, and incubated at 28°C with 5%CO_2 _for 72 hours. Following this incubation period cells were gently rinsed twice with 1 ml of 1×PBS pH7.4, and harvested in 300 μl of 1× passive lysis buffer (Promega). The lysates were spun and the supernatants were moved to new tubes. Ten microliters of the supernatant from each tube was added in triplicate wells of a 96 well microtiter plate in and analyzed using the Dual Luciferase System (Promega) with an LMaxII^384 ^Luminometer (Molecular Devices, Sunnyvale, CA) with the following parameters: 10 μl of each substrate,2 second delay, 5 second reading integration. Firefly luciferase readings were normalized against the *Renilla *luciferase reading by dividing the firefly raw data by the amount of *Renilla *luciferase detected.

Aag2 cells were plated into 9.6 cm^2 ^well in minimal S2 media (Lonza) at a density of 1.0 × 10^6^cells/well. At 15 hours post- plating cells were transfected as performed for S2 cells. Following an overnight incubation (16 hours), cells were washed once with 1 ml Schneider's minimal media and once with 1 ml infection media (Schneider's media containing 2% FBS and 1% essential amino acids). Cells were then overlaid with 2 ml infection media containing DENV-2 at an MOI of 0.01, gently rocked for 1 hr to aid in absorption of the virus, then incubated at 28°C with 5%CO_2 _for 96 hours. Cells were processed and analyzed for luciferase activity as described for the S2 cells above. All luciferase experiments were performed in triplicate.

### TCID_50_-IFA analysis

Assessment of DENV-2 NGC titre was measured using serial 10 fold dilution followed by detection of the cell surface expressed DENV E protein as previously described [4]. Briefly, cell media containing virus from infected C6/36 cells were accumulateded 48 hpi and overlaid onto naive C6/36 cells using 10 fold serial dilutions in a 96 well plate and incubated for 4 days at 28°C without CO_2_. Cells were then fixed with acetone:DPBS (3:1) and stained with a primary DENV envelope (E) antibody (1:200) [50]. Positive DENV-2 NGC infected cells were detected using a biotinylated-streptavidin detection system conjugated with Fluorescein isothiocyanate (FITC; Amersham Biosciences, Piscataway, NJ). Cell cytoplasms displaying fluorescence were scored as positive for DENV infection. The number of positive wells were counted and the virus titers calculated according to Karber's method [51].

## Authors' contributions

JRC engineered the αDENV-GrpI constructs possessing the IRES mCherry configuration and maintenance of DENV-2 NGC viral stocks. JRC also performed all cell culture analysis of the αDENV-GrpI constructs to include dual luciferase and RT-PCR and fluorescence microscopy. JHK engineered the dsDENV-2 NGC fold back construct. JHK and PVB produced the original αDENV-GrpIs used in all analysis. TSF maintained all cell cultures and established all transformed cell lines. The manuscript was prepared predominantly by JRC and MJF with some initial assistance by JHK. All authors read and approved the final manuscript.

## Supplementary Material

Additional file 1 **Sequence composition of the αDENV-GrpI**. The features of each αDENV- GrpI are shown and construction is described in Methods. Right column lists the individual αDENV-GrpIs used in this study. The nucleotide sequences of each region are listed beside the corresponding αDENV-GrpI. Internal guide sequence = IGS, BL = bulge loop, External guide sequence = EGS, P10 = P10 helix. Click here for file

Additional file 2 **Primers and PCR fragments**. The forward and reverse primer sets used to produce the corresponding PCR fragments are listed. Restriction sites are in lower case text. See Methods for description of vector constructs. Click here for file
