# Supplementary figures and images for: Targeting of highly conserved Dengue virus sequences with anti-Dengue virus trans-splicing group I introns
Source: BMC Mol Biol. 2010 Nov 15;11:84. doi: 10.1186/1471-2199-11-84 (PMC3000392; doi:10.1186/1471-2199-11-84)

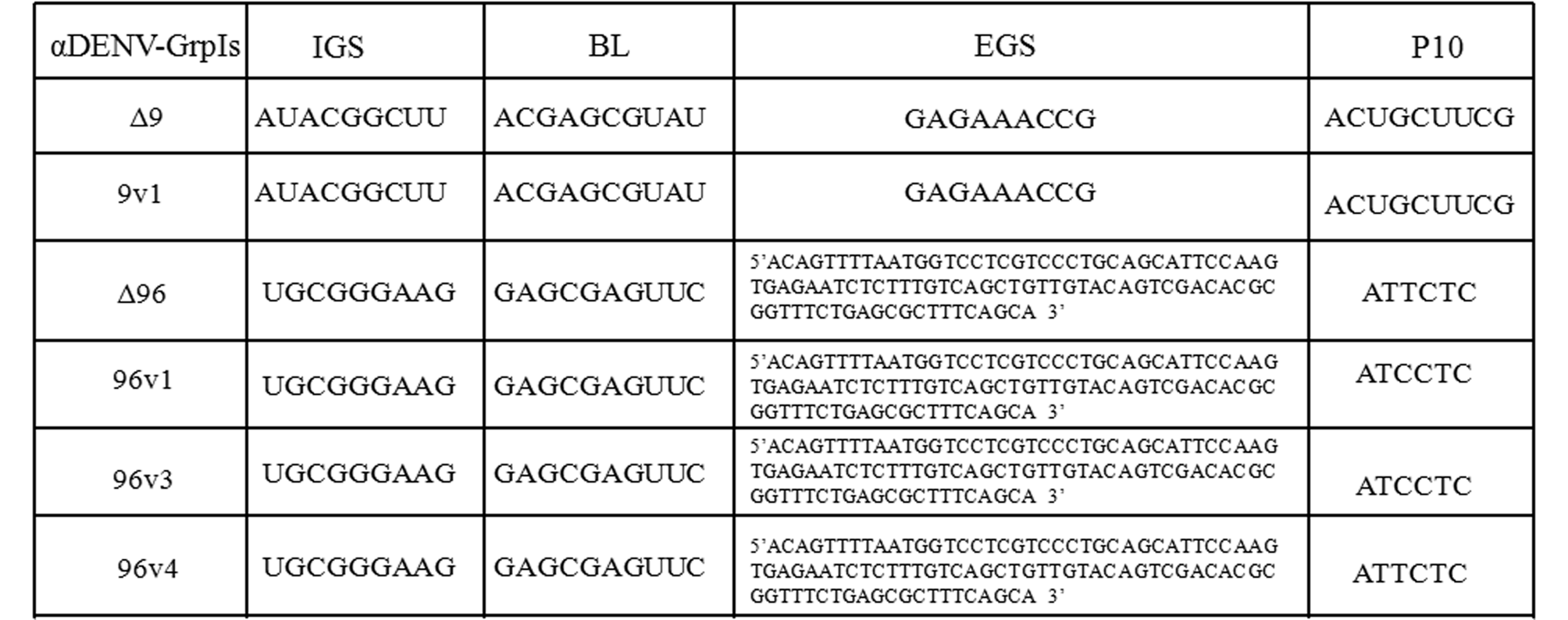

Supplement: Additional file 1 — Sequence composition of the αDENV-GrpI. The features of each αDENV- GrpI are shown and construction is described in Methods. Right column lists the individual αDENV-GrpIs used in this study. The nucleotide sequences of each region are listed beside the corresponding αDENV-GrpI. Internal guide sequence = IGS, BL = bulge loop, External guide sequence = EGS, P10 = P10 helix. [file 1471-2199-11-84-S1.PNG]

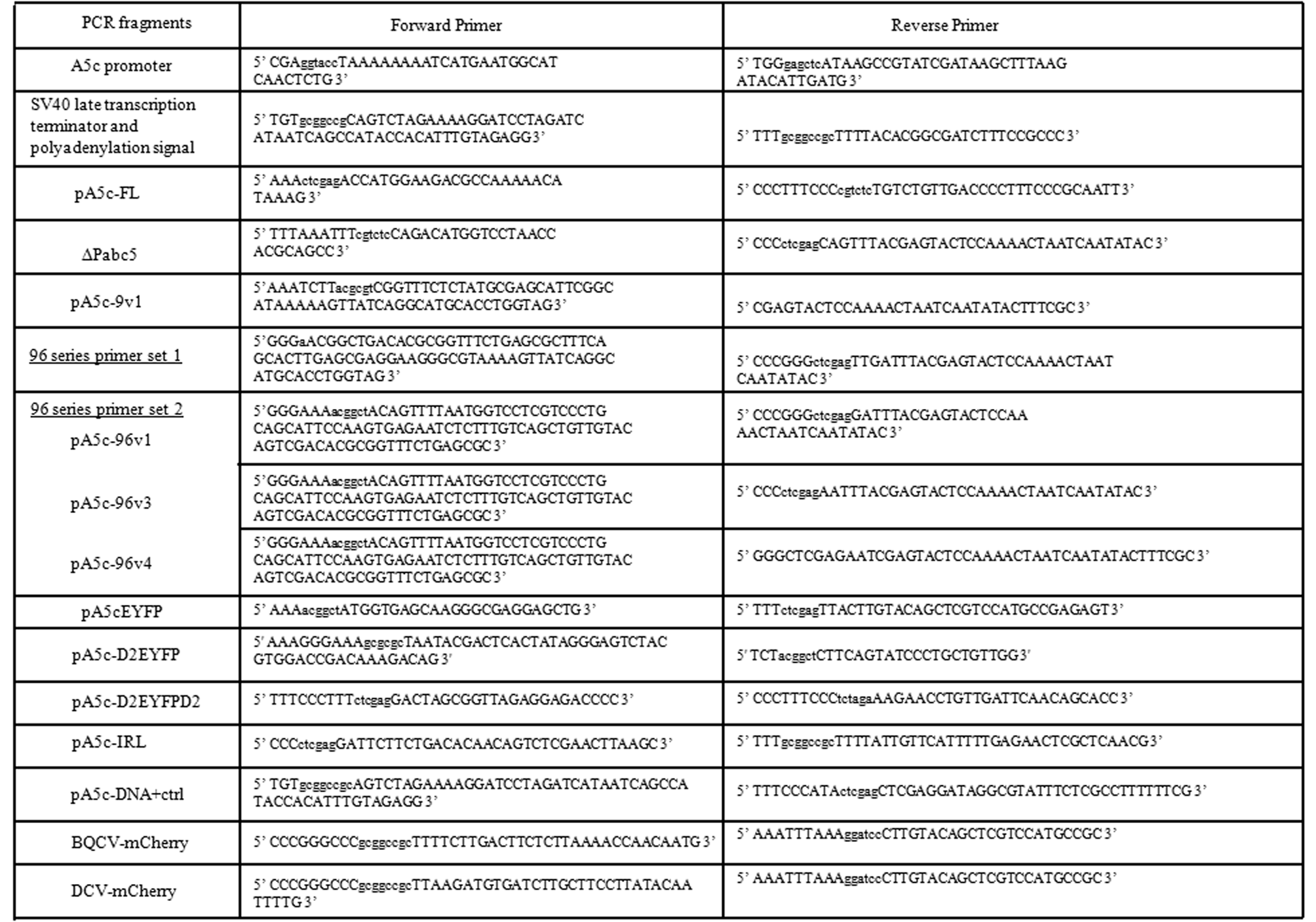

Supplement: Additional file 2 — Primers and PCR fragments. The forward and reverse primer sets used to produce the corresponding PCR fragments are listed. Restriction sites are in lower case text. See Methods for description of vector constructs. [file 1471-2199-11-84-S2.PNG]
